# CT pattern of Infarct location and not infarct volume determines outcome after decompressive hemicraniectomy for Malignant Middle Cerebral Artery Stroke

**DOI:** 10.1038/s41598-019-53556-w

**Published:** 2019-11-19

**Authors:** Saadat Kamran, Naveed Akhtar, Abdul Salam, Ayman Alboudi, Kainat Kamran, Yahiya Bashir Imam, Numan Amir, Musab Ali, Khawaja Hasan Haroon, Ahmad Muhammad, Arsalan Ahmad, Ali Ayyad, Osama Elalamy, Jihad Inshasi, Ashfaq Shuaib

**Affiliations:** 1 Neuroscience Institute (Stroke Center of Excellence), Weil Cornell School of Medicine, Doha, Qatar; 2 Hamad Medical Corporation, Doha, Qatar, Weil Cornell School of Medicine, Doha, Qatar; 3 Rashid Hospital, Dubai, UAE; 4 School of Liberal Arts, University of Illinois, Chicago, USA; 5 Shifa International Hospital, Islamabad, Pakistan; 6 Department of Neurosurgery, Johannes Gutenberg University, Mainz, Germany

**Keywords:** Outcomes research, Outcomes research, Stroke, Stroke

## Abstract

Malignant middle cerebral artery [MMCA] infarction has a different topographic distribution that might confound the relationship between lesion volume and outcome. Retrospective study to determine the multivariable relationship between computerized tomographic [CT] infarct location, volume and outcomes in decompressive hemicraniectomy [DHC] for MMCA infarction. The MCA infarctions were classified into four subgroups by CT, subtotal, complete MCA [co-MCA], Subtotal MCA with additional infarction [Subtotal MCAAI] and co-MCA with additional infarction [Co-MCAAI]. Maximum infarct volume [MIV] was measured on the pre-operative CT. Functional outcome was measured by the modified Rankin Scale [mRS] dichotomized as favourable 0–3 and unfavourable ≥4, at three months. In 137 patients, from least favourable to favourable outcome were co-MCAAI, subtotal MCAAI, co-MCA and subtotal MCA infarction. Co-MCAAI had the worst outcome, 56/57 patients with additional infarction had mRS ≥ 4. Multiple comparisons Scheffe test showed no significant difference in MIV of subtotal *infarction*, co-MCA, Subtotal MCAAI but the outcome was significantly different. Multivariate analysis confirmed MCAAI [7.027 (2.56–19.28), p = 0.000] as the most significant predictor of poor outcomes whereas MIV was not significant [OR, 0.99 (0.99–01.00), p = 0.594]. Other significant independent predictors were age ≥ 55 years 12.14 (2.60–56.02), p = 0.001 and uncal herniation 4.98(1.53–16.19), p = 0.007]. Our data shows the contribution of CT infarction location in determining the functional outcome after DHC. Subgroups of patients undergoing DHC had different outcomes despite comparable infarction volumes.

## Introduction

Notwithstanding the unequivocal survival advantage of DHC, preoperative prediction of functional outcome remains uncertain. Several risk factors that predict functional outcome have been identified but there is considerable variability in the reported factors^1,2^. Though final infarct volume has been reported as a predictor of outcomes in anterior circulation stroke^3^ others have not shown a strong relationship^4,5^. Since MMCA strokes have different topographic distribution and may have associated additional infarction in the anterior cerebral artery [ACA], posterior cerebral artery [PCA] territories, lesion location might confound the relationship between lesion volume and outcome. The purpose of this study was to determine the multivariable relationship between CT infarct location, volume and outcomes in DHC for MMCA infarction. A better prediction of the functional outcome will help the physicians/surgeons and families in deciding the best treatment option for MMCA infarction. Especially, when there is a diversity of opinion among health care personnel, general public and patients regarding acceptable outcome and management of MMCA infarction, despite published evidence and recommendation for DHC^6–8^.

## Material and Methods

In a retrospective, multicenter cross-sectional study data for DHC for MMCA infarction, pooled from 3 tertiary referral centres in 3 countries [Qatar, Pakistan and the United Arab Emirates], collected between 2007 and 2014 was analyzed.

### Inclusion and exclusion criteria

The study included all patients referred for DHC. The patient inclusion criteria were: National Institutes of Health Stroke Scale score [NIHSS] ≥15 including a score of 1 for item 1a (decreased level of consciousness from the beginning or progressive deterioration]. Cerebral computed tomography [CT] showing infarction involving at least two third of the middle cerebral artery territory[MCA] infarction [>50% but <100%] including basal ganglia with or without internal capsule involvement, signs of oedema with or without additional anterior cerebral artery [ACA] or posterior cerebral artery [PCA] involvement.

Data included demographics, risk factors, imaging studies, hyperosmolar treatment, signs of herniation, time of herniation, and time to surgery.

### Radiological data

All radiological measurements were made by consensus between 2 Neuroradiologists blinded to clinical data, and a third opinion [stroke Neuroradiologist] was obtained in cases of disagreement. For determining the lesion pattern and maximum pre-operative infarction volume [MIV] the last CT prior to DHC was used. The infarction volume [IV] was measured by open source image analysis software OsiriX version 5.6^9^. Using a closed polygon tool, lesion outline was marked on the first, middle and last CT slice with ischemic stroke. The software then generates the missing polygons that were checked for accuracy and manually corrected if needed. Once corrected, infarction volume was automatically generated by the software. For temporally separate lesions, each lesion was measured separately and added to generate infarction volume.

For CT stroke patterns we used criteria of the randomized control trials, published literature and templates of Tatu *et al*. as a guide to the topography of stroke by vascular territory^10–16^. The patients were classified into four subgroups according to CT patterns, subtotal *infarction* in the territory of the *middle cerebral artery* [>50% *with basal ganglia involvement]*, complete involvement of the MCA [co-MCA], Subtotal MCA [≥50% MCA] with additional infarction due to ACA and/or PCA involvement [Subtotal MCAAI] and complete MCA with additional infarction due to ACA and/or PCA involvement [Co MCAAI]. The size of the bone flap was measurement included anterior-posterior, vertical measurements from the inner skull table and if middle cranial fossa was opened on postoperative CT scan. For measurement of septum pellucidum shift, a straight line was drawn in the expected location of the septum pellucidum from the posterior-most aspects to the falx on axial images. The shift of the septum pellucidum from this midline was measured on all CT scans and compared to subsequent CT scans to determine any change.

Early signs of uncal herniation on CT scan included medial displacement of the uncus and parahippocampal gyrus of the temporal lobe, encroachment on the suprasellar cistern, narrowing of the contralateral ambient and quadrigeminal plate cisterns with the ipsilateral widening of the ambient and quadrigeminal cisterns^17^.

Patients with only a single imaging study, parenchymal hematoma grade II^18^, haemorrhage with ventricular extension or missing surgical details were excluded from the analysis.

The decision for DHC was made by the treating physicians/neurosurgeons based upon the individual clinical condition and cerebral CT imaging. Patients were generally taken to surgery if their level of consciousness was progressively deteriorating with or without early clinical signs of herniation. All patients underwent hemicraniectomy and duroplasty without removal of the infarcted tissue. The size of the bone flap and opening of the middle cranial fossa was at the discretion of the surgeon. Surgical and postoperative complications were recorded. The bone flap was replaced within 3 months of surgery.

The primary outcome was measured using a modified Rankin Scale (mRS) score at three months by patient examination in the outpatient clinics. Clinical outcome was categorized as favourable (mRS 0–3) and (mRS 0–4) or unfavourable (mRS 4–6) and (mRS 5–6) based on post hoc analysis of the pooled data of the randomized control trials^19^.

All the tertiary referral centres in the study have a well-established comprehensive stroke service including stroke diagnostic, vascular interventional, stroke units, neurosurgical and rehabilitation services. An acute stroke team is available 24 hours a day, seven days a week.

Because of retrospective data evaluation the informed consent was waived by the Institutional Review Board of Hamad Medical Corporation, Qatar, Shifa International Hospital, Pakistan and Rashid Hospital, UAE.

### Data analysis

All statistical analyses were performed using Statistical Package for Social Sciences Version 22 (SPSS). Descriptive and inferential statistics were used to characterize the study sample and test hypotheses. Descriptive results (including graphical displays) for all quantitative variables (e.g., age) are presented as mean ± standard deviation (SD) (for normally distributed data) or median with interquartile range (for data not normally distributed). Numbers (percentage) were reported for all qualitative variables (e.g., gender). Bivariate analysis was performed using ANOVA, and Kruskal-Wallis test to compare the average for all quantitative variables (e.g., age) among types of infarct location (subtotal MCA *infarction*, co-MCA, Subtotal MCAAI and Co-MCAAI). Whenever appropriate, while Pearson Chi-Square test or Fisher Exact test as appropriate were used to comparing all the qualitative variables (e.g. gender) among types of the infarct. Multiple comparisons analysis were performed using the Scheffe test if there was an overall statistically significant difference for the average of maximum infarct volume and mRS at three months using One Way ANOVA. Multiple logistic regression models were used to identify significant independent factors associated with functional outcome (mRS 0–4 vs. 5–6) at three months after adjusting for potentially confounding factors. To build the model, a purposeful selection method was used for selecting a subset of covariates considered clinically important, adjusting for confounders and statistical significance. Purposeful selection of covariates begins with a multivariate model that contains all variables that are significant in the bivariate analysis at the 20–25 per cent level, as well as any other variables not selected with this criterion but judged to be of clinical importance. We used p-values from the Wald tests of the individual coefficient to identify covariates that might be deleted from the model and p-value of the partial likelihood ratio test confirming that the deleted covariate is not significant. Following the fitting of the reduced model, we assessed whether or not a removal of the covariate will produce an “important” change (about 20%) in the coefficient of the variables remaining in the model. The final model was assessed using the Hosmer-Lemeshow Goodness-of-fit statistics to determine whether the model adequately describes the data. Adjusted Odds ratio and 95% confidence interval for the adjusted odds ratio were reported for each independent predictor. A “P” value < 0.05 (two-tailed) was considered statistically significant.

### Compliance with ethical standards

The study adhered to the tenets of the declaration of Helsinki and was approved by the Institutional Review Board of Hamad Medical Corporation, Qatar [15246/15], Shifa International Hospital, Pakistan 421-270-2014, and Rashid Hospital, UAE DSREC 12/2015_09.

## Results

Out of two hundred and thirty-two patients, 146 patients underwent DHC, 67 patients selected for DHC did not undergo surgery [19 who refused surgery expired and 48 stabilized and the treating surgeon decided not to operate]. Nine patients were excluded from the DHC analysis (incomplete data, 2; haemorrhage with ventricular extension, 4; haemorrhage causing acute worsening, (PH II) 3). In the final analysis, 137 patients who underwent DHC were included.

The demographics, clinical features and risk factors are summarized in Table 1.

**Table 1:** Relationship between demographic, clinical characteristics and MCA with and without additional infarct.

Factors	Total (n = 137)	Subtotal MCA (n = 28)	Co MCA (n = 52)	Subtotal MCAAI (n = 25)	Co MCAAI (n = 32)	P-value
Age	47.88 ± 11.00	45.14 ± 10.99	47.88 ± 10.30	48.28 ± 8.06	49.97 ± 13.76	0.236
<55years	104(75.9%)	25(89.3)	41(78.8)	20(80.0)	18(56.3)	0.019
≥ 55 years	33(24.1%)	3(10.7)	11(21.2)	5(20.0)	14(43.8)	
Gender						0.313
Male	111 (81.0%)	24(85.7)	38(73.1))	22(88.0)	27(84.4)	
Female	26 (19.0)	4 (14.3)	14 (26.9)	3(12.0)	5(15.6)	
**Risk Factors**						
Hypertension	63 (46.0)	15 (53.6)	19 (36.5)	13(52.0)	16(50.0)	0.378
Diabetes	47 (34.3)	10 (35.7)	14 (26.9)	7(28.0)	16(50.0)	0.156
Dyslipidemia	50 (36.5)	9 (32.1)	20 (38.5)	9(36.0)	12(37.5)	0.954
CAD	30 (21.9)	5 (17.9)	10 (19.2)	6(24.0)	9(28.1)	0.735
Atrial Fibrillation	13 (9.5)	3 (10.7)	2 (3.8)	4(16.0)	4(12.5)	0.315
CHF	12 (8.8)	2 (7.1)	3 (5.8)	3(12.0)	4(12.5)	0.668
Arrival Time (hours)	4.49 ± 5.38	4.52 ± 5.23	5.52 ± 6.38	4.17 ± 5.47	3.05 ± 2.97	0.482
Uncal Herniation on CT	92(67.2)	2(7.1)	52(100.0)	6(24.0)	32(100.0)	<0.001
Subfalcine Herniation on CT	39(28.5)	6(21.4)	14(26.9)	7(28.0)	12(37.5)	0.567
Comatose	83(60.6)	17(60.7)	32(61.5)	14(56.0)	20(62.5)	0.962
Pupillary abnormality	27(19.7)	2(7.1)	10(19.2)	4(16.0)	11(34.4)	0.061
Bilateral Babinski	83(60.6)	15(53.6)	33(63.5)	15(60.0)	20(62.5)	0.847
Herniation time from onset						0.009
<24 hours	29(21.2)	1(3.6)	14(26.9)	4(16.0)	10(31.3)	
24-<48 hours	51(37.2)	6(21.4)	18(34.6)	14(56.0)	13(40.6)	
48-<72 hours	30(21.9)	11(39.3)	12(23.1)	3(12.0)	4(12.5)	
≥72 hours	27(19.7)	10(35.7)	8(15.4)	4(16.0)	5(15.6)	
SP displacement last CT ≥ 1 cm	81(59.1)	16(57.1)	28(53.8)	15(60.0)	22(68.8)	0.598
CT time to MIV (hr.)	58.06 ± 55.79	85.58 ± 84.96	48.44 ± 32.40	47.25 ± 31.90	58.05 ± 62.60	0.044
MIV (cm^3^)	367.7 ± 119.44	304.9 ± 97.15	367.7 ± 113.7	347.6 ± 110.5	438.2 ± 120.9	<0.001
DHC Time						
≤48 hours	54 (39.4%)	3(10.7%)	21(40.4%)	11(44.0)	19(59.4)	0.002
>48 hours	83 (60.6%)	25(89.3%)	31(59.6%)	14(56.0)	13(40.6)	
Centers involved						
Doha- Qatar	Dubai-UAE	88[64.2%]	18 [63.45%]	34[65.4%]	18[72.0%]	18[56.3%]
Doha- Qatar	39[28.5%]	8 [28.6%]	12[23.1%]	6[24.0%]	13[40.6%]	
I Islamabad-Pakistan	10[7.37]	2 [7.1%]	6[11.5%]	1[4.0%]	1[3.1%]	
**Prognosis at three month**						
Favorable (mRS0–3)	34(24.8)	14(50.0)	19(36.5)	1(4.0)	0	<0.001
Unfavorable (mRS 4–6)	103|(75.2)	14(50.0)	33(63.5)	24(96.0)	32(100.0)	
Favorable (mRS0–4)	84(61.3)	28(100.0)	35(67.3)	11(44.0)	10(31.3)	<0.001
Unfavorable (mRS 5–6)	53|(38.7)	0	17(32.7)	14(56.0)	22(68.8)	
30 Day Mortality	23(16.8)	0	4(7.7)	8(32.0)	11(34.4)	<0.001

*Results are expressed as mean* ± *standard deviation, Median (Inter Quartile Range), and number (percentage), Median (Inter Quartile Range), and number (percentage)*. MIV Maximum Infarct Volume, SP septum pellucidum.

Out of 137 patients, only 57 (41.6%) had ipsilateral additional infarctions [55 ACA, 1PCA and 1ACA + PCA], while 80 (58.4%) had MCA strokes without any additional infarctions. In univariate analysis, patient with MCA without additional infarction was younger [p = 0.019]. There were more males due to the skewed male predominant population of expatriates in Qatar and UAE. No difference was observed in admission NIHSS [p = 0.083], preoperative GCS [p = 0.118], risk factors, use of anti-edema therapy [p = 0.236], length of ventilation [p = 0.804] and sub-falcine [p = 0.567] herniation. More patients with co-MCAAI showed signs of herniation in less than 48 hours [p = 0.009]. Though mean infarct volume was higher in patients with co-MCAAI [0.010] there was no difference in septum pellucidum displacement on preoperative [last] CT [p = 0.598]. Meantime to DHC was 51.33 hours (range: 12 to 312 hours). More patients with co-MCAAI was operated in <48 hours [p = 0.002], time to surgery had no impact on the outcome at three months [P = 0.109]. There was no difference in the postoperative complications, development of subgleal hematoma >1 cm [0.235], aspiration pneumonia [0.189], urinary tract infection [0.296] and seizure [0.140]. Mean anterior-posterior and vertical bone flap measurement [P = 0.560] and opening of the middle cranial fossa [P = 0.745] had no significant impact on the outcome.

There was no significant difference in the infarction pattern between the participating centres [p = 0.50].

### Multiple comparisons scheffe test

**Figure 1:**
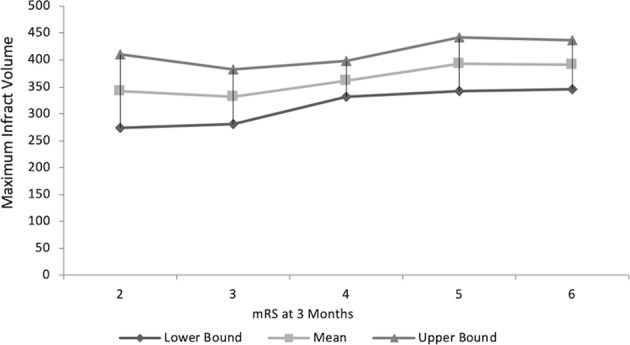
Maximum preoperative Infarct volume with mRS at 3 months (Results expressed as MIV with 95% Confidence Interval for each mRS score at three months) indicating no significant difference [p = 0.295].

The relationship between types of the infarct, maximum infarct volume and mRS at three months is shown in Table 2, Fig. 1.

**Table 2:** Multiple comparisons Scheffe test. Relationship between infarct topography and Maximum infarct volume, mRS score at three months.

Type of Infarct	MIV cm3	3-month mRS
Subtotal MCA infarction	304.98 ± 97.15^a^	3.39 ± 0.68^a^
Complete MCA infarction	367.70 ± 113.73^b^	3.94 ± 1.11^b^
Subtotal MCA with additional infarction	347.64 ± 110.48^c^	4.84 ± 0.94^ab^
Complete MCA with additional infarction	438.25 ± 120.94^abc^	5.03 ± 0.82^ab^
P-value	<0.001	<0.001

^a^2/3rd MCA is different, ^b^Complete MCA is different, ^c^2/3rd MCA with add. Infarct is different. Results are expressed as Mean ± Standard Deviation.

MIV of co-MCAAI is significantly different from (Subtotal MCA or co-MCA or Subtotal MCAAI). There was no statistical difference in the mean MIV among the three types of infarction (Subtotal MCA or co-MCA or Subtotal MCAAI). Average mRS between Subtotal MCA and co-MCA was not different while MCA with additional infarction [co-MCA or Subtotal MCA] was significantly different from Subtotal MCA and co-MCA without additional infarction. There was no difference between both additional infarction groups.

### MIV (cm^3^)

ANOVA test revealed a statistically significant difference in the mean maximum infarction volume among the types of infarction (p-value < 0.001). Multiple comparisons Scheffe test indicated that MIV in patients with co-MCAAI is significantly different from other types of infarction (subtotal MCA, co-MCA and Subtotal MCAAI). There was no statistical difference in the mean MIV among the other three types of infarction (subtotal MCA, co-MCA and Subtotal MCAAI) Tables 1,2, Fig. 1.

### Functional outcome [mRS at 3 months]

ANOVA test revealed that there was a statistically significant difference in the mRS at three months among the types of infarct (p-value = 0.001). Multiple comparisons Scheffe test indicate that mRS at three months in patients with subtotal MCA is significantly different from patients with Subtotal MCAAI and patient with co-MCAAI. Similarly, mRS at three months among patient with co-MCA is significantly different from patients with subtotal MCAAI and patient with co-MCAAI. But there was no statistically significant difference in mRS at three months between patients with subtotal MCA and co-MCA.

Despite no statistically significant difference in MIV, mRS at three months in co-MCA infarction was significantly different from subtotal MCAAI and patient with co-MCAAI. Average mRS between subtotal MCA and co-MCA was not significantly different while subtotal MCAAI and co-MCAAI was significantly different from Subtotal MCA and co-MCA. There was no difference between the subtotal MCAAI and co-MCAAI.

**Figure 2:**
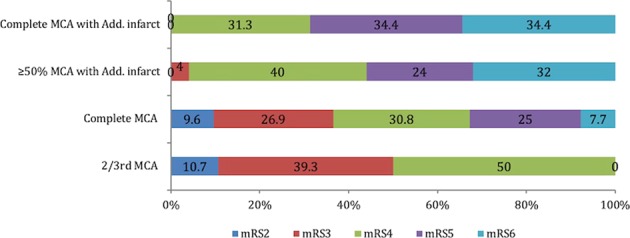
Outcome at three months according to infarction topography, p < 0.001.

The functional outcome was better in MCA stroke [subtotal and complete] [p = 0.001] [Table 1, Fig. 2]. Most patients in all subgroups had an mRS of 4, irrespective of CT infarction location except co-MCAAI where it was evenly split between mRS 5 or 6. Only one patient with subtotal MCAAI survived with an mRS of 3 out of 57 patients with additional infarction. None of the patients with subtotal MCA infarction had an mRS of 5 or 6. The highest mortality was observed in the subtotal MCAAI and co-MCAAI groups [19/23, 82.60%; p = 0.001]. All deaths happened within two weeks of stroke onset as a result of transtentorial herniation.

### Multiple logistic regression analysis

Multiple logistic regression analysis [Table 3] confirmed co MCAAI 7.027 (2.56–19.28), p = 0.000] as independent predictor of functional outcomes at three month after adjusting for confounders [age, gender, MIV, uncal Herniation on CT, SP displacement ≥1 cm, participating sites, hypertension, diabetes, dyslipidemia, CAD, atrial fibrillation, congestive heart failure, NIHSS at admission, intracerebral hemorrhage at the craniotomy site, subgleal and subdural hematoma, infection at the craniotomy site, aspiration pneumonia, and seizures. The MIV did not show any significant association [0.99 (0.99–01.00), p = 0.594] in the presence of MCA with additional infarction after adjusting for confounders.

**Table 3:** Multivariate logistic regression model to confirm MCA with additional infarction is a significant independent predictor for functional outcomes at three months.

Factors	Adjusted Odds ratio (AOR) (95% CI for AOR)	P-value
MCA with additional infarct	6.54 (2.31–18.47)	<0.001
Uncal Herniation on CT	4.86(1.39–16.89)	0.013
Age ≥ 55 years	14.95 (2.87–77.91)	0.001
SP displacement ≥1 cm	2.78 (0.91–8.51)	0.073
Max Infarct Volume	0.999 (0.99–1.01)	0.643
**Participating centres**		
Islamabad-Pakistan	1	0.212
Dubai-UAE	7.71[0.35–167.89	0.194
Doha-Qatar	14.22[0.65–308.58	0.091
Gender [male]	2.94[0.70–12.33]	0.140
Hypertension	1.74[0.55–5.51]	0.343
Diabetes	2.19[0.68–7.02]	0.186
Dyslipidemia	0.929[0.29–3.01]	0.902
Coronary artery disease	0.28[0.06–1.33]	0.110
Atrial fibrillation	0.38[0.038–3.85]	0.415
Congestive heart failure	2.55[0.31–21.27]	0.388
Admission NIHSS	1.101[0.96–1.26]	0.157
ICH at craniotomy site	0.59 (0.12–2.90)	0.513
Subgleal, Subdural hematoma	1.68 (0.29–9.54)	0.558
Aspiration pneumonia	1.51 (0.38–6.05)	0.558
Infection craniotomy site	0.89 (0.21–3.72)	0.871
Seizures	1.63 (0.20–13.02)	0.646

CI: Confidence Interval; P-value has been calculated using binary multiple logistic regression Wald test.

ICH intracerebral hemorrhage.

## Discussion

Our data support the hypothesis that the pattern of CT infarction location and not the MIV is the major determinant of functional outcome after DHC. Subgroups of patients undergoing DHC had different outcomes despite comparable infarction volumes but differed in infarction location. The stroke groups could be arranged in order of the most to least unfavourable functional outcome after DHC were co-MCAAI, Subtotal MCAAI, co-MCA and subtotal MCA infarction. MMCA with additional infarction involving ACA and/or PCA had the worst functional outcome after DHC.

With similar MIV, co-MCA and subtotal MCAAI had significantly different functional outcomes [Fig. 2]. Interestingly, direct physical factors, such as involvement of ACA and/or PCA arteries apart from MCA, the extent of midline shift and CT signs of uncal herniation bore a significant relationship to the functional outcome. In fact, the outcome was unfavourable if there was any additional stroke [ACA, PCA] irrespective of the size of MCA infarction [Fig. 2]. In HAMLET trial 34% of patients in the control arm had MCAAI compared to 22% who underwent surgery, with increased mortality and unfavourable outcome in the control arm^13^. The ACA and PCA provide important collateral blood flow to the conventionally defined MCA territory and therefore ACA and/or PCA ischemia is likely to be detrimental to the perfusion of the various MCA territories as well. The presence of additional infarcts [ACA, PCA] not only increases the infarction volume and cerebral oedema^20^ but add to the cognitive, behavioural impairments and aggravate the functional deficits, thereby, compromise the potential for recovery^21–23^.

We accounted for infarction location, infarction volume, as well as clinical characteristics to develop multivariable risk adjustment models, identifying predisposing factors associated with an unfavourable functional outcome. Although it is reasonable to assume that MCA with additional infarction will have lager infarction volumes and therefore, the poor functional outcome as reported recently^24^. In this study, most of the cohort had MCA with additional strokes [49/92; 53.26%] leading to higher infarct volume thus relating poor functional outcome to infarction volume. Amongst the randomized trials of DHC, only DECIMAL used infarction volume in addition to the involvement of MCA >50% for enrolment in the study^10^. Similar to the findings of the DECIMAL trial of a non-significant trend towards a poor outcome with higher infarct volumes at inclusion our outcome regression prediction model did not find any significant relation of MIV to functional outcome. It is also possible that CT infarction location [MCA with additional infarction] covaried with MIV, explaining why MIV was not a significant predictor in the regression analysis.

The current study has several limitations including the retrospective nature and the lack of a randomized comparison between groups. We used CT scan lesion patterns to classify patients into groups. The lack of detailed vascular imaging and status of collateral circulation on all patients may have led us to misclassify a complete MCA [due variation in the circulation with ACA and PCA feeding a part of MCA territory] as a subtotal MCA stroke. However, CT lesion patterns [>50% of the MCA territory, DECIMAL and HeADDFIRST, and 2/3 of the territory and including at least part of the basal ganglia, DESTINY and HAMLET] was used in randomized trials of DHC. The data was collected from hospitals in three countries and reflects real life experience rather than clinical trials. There were no local guidelines for DHC patient referral and pre-operative care was not standardized between the participating centres that could have influenced patients’ selection and timing of surgery. Moreover, selection bias due to the difference in criteria for surgical selection between treating physicians/surgeons and centres with widely applied standardization protocol for DHC cannot be excluded. Although we have shown that infarction location and not MIV is important but there is a possibility that due to smaller sample size in subgroups there was lack of statistical power to resolve the association of MIV vs. location and outcome. The predominant male population in our study was due to the males expatriate population in the UAE and Qatar. Finally, a major limitation is the short-term (3-month) follow-up and type of rehabilitation received. As most expatriates will leave the country [Qatar and UAE] after treatment, long-term follow-up was not available.

The present study shows the contribution of infarction location to the outcome after DHC. With the same MIV, infarction location showed wide variations in the outcome highlighting the difficulty in outcome prediction. The predictive value of preoperative CT infarction location may help in the decision-making process in the identification of patients at risk of poor functional outcomes, enhance our current strategies of patient selection for DHC and assist in the planning of rehabilitation. Our study is relevant in the context of increasing stroke burden in low and middle-income countries^25^ where modern intervention techniques [thrombolysis, thrombectomy] are either too expensive or not available.
